# Novel Ag-modified zirconia nanomaterials with antibacterial activity

**DOI:** 10.1039/d5ra07099f

**Published:** 2026-01-09

**Authors:** Gabriel Onyenso, Jiwar AI-Zawity, Nastaran Farahbakhsh, Annika Schardt, Aydan Yadigarli, Swathi Naidu Vakamulla Raghu, Carsten Engelhard, Mareike Müller, Holger Schönherr, Manuela S. Killian

**Affiliations:** a Chemistry and Structure of Novel Materials, University of Siegen Paul-Bonatz-Str. 9-11 57076 Siegen Germany; b Physical Chemistry I, University of Siegen Adolf-Reichwein-Str. 2 57076 Siegen Germany; c Analytical Chemistry, University of Siegen Adolf-Reichwein-Str. 2 57076 Siegen Germany; d Federal Institute for Materials Research and Testing (BAM) Richard-Willstätter Str. 11 D-12489 Berlin Germany; e Research Center of Micro- and Nanochemistry and (Bio)Technology (Cµ), University of Siegen Germany

## Abstract

The outcome of an implant procedure largely depends on the implant's surface properties. Biomaterials are now designed to have surfaces with multifunctionality, such as favorable tissue integration and the ability to combat bacterial adhesion and colonization. Herein, we report on a simple approach to improve the antibacterial properties of zirconia nanotubes (ZrNTs) coatings by decorating with silver nanoparticles (AgNP), achieved through electrochemical anodization of a zirconium–silver alloy (Zr–Ag). The AgNPs were shown to partially consist of Ag_2_O, potentially enhancing the availability of Ag^+^ ions for antibacterial activity. The modified ZrNTs were characterized using SEM, EDS, ToF-SIMS, and XPS to determine their structural morphology and chemical composition, and were further subjected to antibacterial testing. The silver and zirconium ion release behavior was monitored *via* ICP-MS. ZrNTs decorated with AgNP exhibit strong antimicrobial activity (>99% bacterial killing) against both *S. aureus* and *E. coli.* Antimicrobial tests indicate that the antibacterial activity against the Gram-positive pathogen *S. aureus* was improved by a factor of 100 compared to unmodified ZrNTs, while unmodified ZrNTs already showed a comparable reduction of viable Gram-negative *E. coli*. This strategy illustrates a straightforward and effective modification that optimizes the interface between the host environment and the biomaterial surface to meet the very important criteria of biocompatibility and active antibacterial response.

## Abstract 2

Ag-incorporated zirconia nanotubes fabricated by anodizing Ag–Zr alloy, for enhanced antibacterial properties.

## Graphical abstract

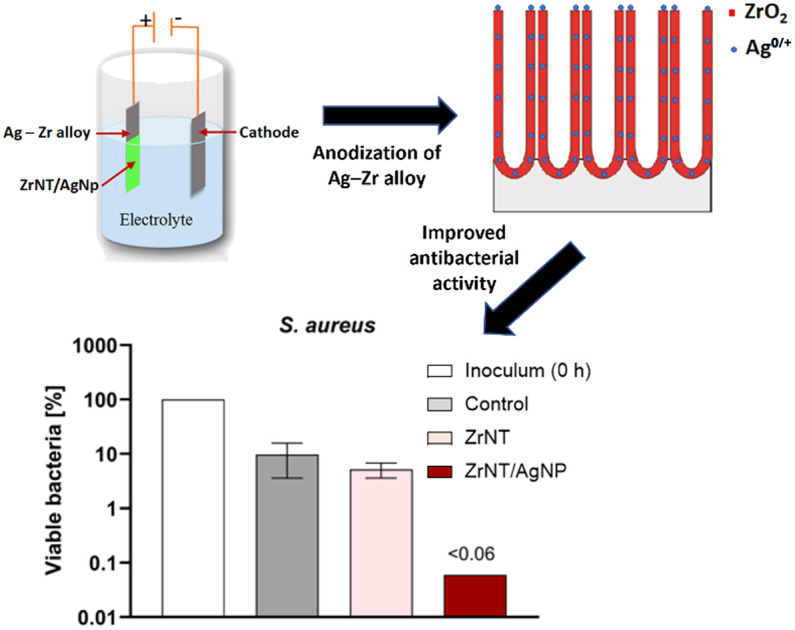

## Introduction

The surface properties of an implanted biomaterial are crucial to its biocompatibility.^1–3^ Biocompatibility has been the fundamental requirement for every biomaterial, and initially, this refers to the ability of a material to be biologically inert.^4,5^ However, this precondition is no longer sufficient in light of the recent endeavors to extend the utility of biomaterials from being just a replacement tool to bioactive systems capable of tissue engineering.^6^ Furthermore, the challenges associated with the use of biomaterial implants, such as biomaterial-associated infections and lack of native tissue integration, depend largely on the chemical and physical properties of the implant surface.^7,8^ The latter is often associated with biofilm formation on the implant or the surrounding tissue with *Staphylococcus aureus* (*S. aureus*) as one of the common causative pathogens, especially in orthopedic applications.^9^ It has been shown that the requirement to inhibit infection and to promote integration of tissue is not mutually exclusive, and there is a link between the ability of an implant surface to resist bacterial adhesion and its capacity to encourage tissue integration and even stimulate specific cellular responses.^10–12^ The phrase “race for the surface” describes the fate of a successful implant procedure (*i.e.*, the possibility of preventing biomaterial-related infections) in terms of a contest between the host tissue cell integration and bacterial adhesion to the implant surface. This concept, largely credited to Anthony Gristina,^13^ is now widely accepted in the field of biomaterials. It relates incidents of biomaterial-associated infections and/or the lack of tissue integration to the initial bacterial colonization of the implant surface.^14,15^ This theory has been experimentally studied, and it was demonstrated that the competition for attachment to the implant surface between desirable host cells and bacteria depends strongly on the amount of the initial bacteria on the surface.^16^ Therefore, biomaterials with elaborately modified surfaces capable of multifunctional abilities have been designed to resist bacterial adhesion while simultaneously promoting host cell integration.^17,18^

Surface modification of implant materials is not a recent endeavor; simple techniques like mechanical polishing, grinding, and blasting, which introduce some form of topographical changes with micrometric features, have been investigated for their ability to improve osseointegration.^19–21^ These procedures, while being a straightforward micro/macro-scale approach, are limited in their efficacy, as it has been shown that interaction between a biomaterial and host tissue occurs additionally at the nanoscale. Therefore, control can further be optimized by nanoscale modification.^22,23^ An effective method for nanoscale modification of metallic biomaterials is by coating with nanoporous/nanotubular structures of the corresponding metal oxides, *e.g.*, by anodization. Such structures have received attention due to their chemical stability, good wettability, mechanical strength, and biocompatibility.^24–27^ Additionally, such nanostructures allow the incorporation of multifunctionality, as the inner volume can be loaded with various therapeutic agents, including antibiotics, that can be released in a controlled manner. Furthermore, nanoscale morphology has been shown to affect cell behavior as it can be modified to selectively attach to a desired host tissue.^28–31^ These nanostructures can be fabricated by a relatively simple, straightforward electrochemical anodization in a fluoride-containing electrolyte.^32–34^ Nanoporous/nanotubular structures of titania and zirconia can be synthesized *via* anodization with good control over their dimension and geometry. They have previously been investigated as a potential drug delivery coating on implants.^28,35,36^ Titanium and zirconium as well as zirconia based materials have already been widely used in fabricating implant devices, mostly for orthopedic and dental applications due to their mechanical strength, good corrosion resistance, and biocompatibility.^37–39^ Attempts at enhancing the antibacterial properties of zirconia and titania nanotubes (ZrNTs and TiNTs) coatings entailed multiple-step approaches of initially fabricating the nanotube before loading with antibiotics and subsequent release studies.^40–42^ These various studies have shown high potential, for example, Popat *et al.* loaded titania nanotubes with gentamicin and showed a reduced bacterial adhesion and enhanced osteoblast differentiation on the nanotubes filled with gentamicin.^42^ However, the drawback of simply loading with antibiotics that are mostly physisorbed on nanotubes is the problem of toxicity, sometimes due to initial burst release, a characteristic release behavior from such systems, and a lack of long-term antibacterial properties. Efforts to impart a seemingly longer antibacterial effect have involved additional steps of coating with biopolymers^43^ or polymer brushes.^44^ While this approach can increase the release time, there is the worry of introducing extra material in the fabrication process, which in addition to increased cost, and differences in material properties (*i.e.* mechanical strength) can restrict its application. Furthermore, there is always the problem of degradation of the polymer over time.^45^ Loading with silver nanoparticles has also attracted interest, as the use of organic antibiotics has the problem of a limited range of antimicrobial activities and, in some instances, is ineffective due to antibiotic resistance development.^46,47^ Silver (Ag) salt and nanoparticles have demonstrated a broad spectrum of antibacterial properties and are currently being used in a variety of medical devices/materials to prevent bacterial infections.^48–50^ Several techniques, such as chemical reduction,^51^ silanization,^52^ photo-reduction^53^*etc.*, have been investigated for incorporating Ag within nanotubes. While these methods enhanced the antibacterial properties of the nanomaterial, there have been reports of cytotoxicity with such systems due to the high initial release of Ag nanoparticles (AgNPs). Furthermore, these methods require a longer development time and can be complex; in particular, the Ag loading process also requires optimization.^54,55^

In this work, an alternative strategy for decorating nanotubes with Ag, that avoids the multi-step process of nanotube fabrication before externally loading with Ag salts, preserves the nanostructural morphology that has been shown to facilitate cellular integration and, importantly, exhibits antibacterial properties with a very low amount of Ag. This facile method involves the electrochemical anodization of zirconium/silver alloy. Previously, Ti–Au alloys have been successfully anodized to achieve site-selective Au decoration of titania nanotubes for photocatalytic hydrogen evolution.^56^ Here, a custom-made Zr–Ag alloy, consisting of silver metal (∼1 wt%) homogeneously mixed with zirconium metal, was anodized at constant voltage conditions in a fluoride-containing electrolyte to obtain a composite material with zirconia nanotubes embedded with silver oxide and AgNP on the surface and along the length of the nanostructure. These hybrid nanostructures were characterized *via* SEM, XPS, and ToF-SIMS, and tested for their antibacterial properties. The intended application of the antibacterial AgNP-ZrNT structure is as a coating for zirconium-based devices.

## Experimental

### Fabrication of ZrNT/Ag_2_O nanotubes decorated with silver nanoparticles

Zirconium/silver alloy foil (∼1 wt% Ag, 0.1 mm thickness, HMW Hauner, Germany) before electrochemical anodization was ultrasonicated in acetone, ethanol, and deionized water for 10 min each and dried under a N_2_ stream. The anodization was done using a high-voltage potentiostat (Jaissle IMP 88 – 200 PC) with an electrochemical cell consisting of a circular working area of 1 cm^2^ and a platinum counter electrode in a typical two-electrode setup. The organic electrolyte used in this experiment is based on the previous work of Vakamulla *et al.* and contains 2 wt% NH_4_F (Sigma-Aldrich), 1 wt% H_2_O, and 30% v/v formamide (Carl Roth) in glycerol (Carl Roth).^24^ The Zr–Ag alloy was anodized for 1 h at 90 V constant voltage after a gradual ramping in 60 s. The current density and voltage during anodization were recorded and monitored by the ECM-Win software. The as-anodized ZrO_2_/AgNP nanotubes were rinsed with deionized water and soaked in ethanol for 10 min to dissolve remains from the organic electrolyte, and then dried in N_2_. The non-modified ZrNTs arrays were prepared using Zirconium foil with the same anodization steps.

### Sample characterization

The surface and cross-sectional morphology of the anodized samples were determined by using scanning electron microscopy (SEM; Quanta FEG 250 FEI) at low vacuum and 30 kV accelerating voltage. The cross-section image was obtained by cutting the anodized foil through the middle to expose the edge of the anodized area and imaging this edge by using a sample holder at 90° tilt. Energy dispersive X-ray spectroscopy (EDS) at 30 kV, which is coupled to the SEM, was used to determine the elemental composition of the samples. X-ray photoelectron spectroscopy (XPS/ESCA; SSX-100 S-probe), which also gives information about the surface chemical components, was performed with monochromatized Al K_α_ radiation, and the core level binding energies were normalized using the adventitious C 1s peak set at 284.8 eV. Time of flight secondary ion mass spectrometry (ToF-SIMS), which also provides surface chemical information, was conducted in positive and negative polarity on a ToF-SIMS 4 instrument (IONTOF GmbH, Münster, Germany) using a 25 keV Bi^+^ ion beam bunched down to <0.8 ns. A minimum of 5 spots were measured per sample and polarity; the primary ion dose density (PIDD) was kept at 1 × 10^11^ ions × cm^−2^, ensuring static conditions. Spectra were calibrated to the CH_3_^+^, C_2_H_3_^+^, C_3_H_5_^+^, C_4_H_9_^+^ and C_7_H_7_^+^ peaks and CH_2_^−^, C_2_^−^, CN^−^ and CNO^−^ peaks, respectively.

### Ag^+^ release studies

To investigate the Ag release characteristics of our samples, they were immersed in 10 mL of phosphate-buffered saline (PBS) at 37 °C. After 24 h storage in the dark, the complete solution was removed for analysis, and 10 mL fresh PBS was transferred back into the container with the sample substrates. The release from the samples was monitored for 10 days to mimic the physiological conditions inside the human body. The obtained immersion liquids of each day were stored in the fridge until the day of measurement. All samples were analyzed on the same day. The released ions were quantified using inductively coupled plasma mass spectrometry (ICP-MS) on a model iCap Qc ICP-Q-MS (Thermo Fisher Scientific, Bremen, Germany). The Qtegra ISDS software (2.10.3324.131, Thermo Fisher Scientific) was used to control the instrument. Sample introduction was achieved with a model ESI SC-2 DX autosampler (ESI Elemental Service & Instruments GmbH, Mainz, Germany), a MicroFlow PFA-ST nebulizer (Thermo Fisher Scientific) with a sample flow rate of *ca.* 470 µL min^−1^, and a Peltier-cooled cyclonic quartz spray chamber (cooled to 3 °C). The plasma torch inner diameter was 1 mm, and the sampling position was set to 5 mm. The high-sensitivity skimmer cone insert “2.8” (Glass Expansion, Melbourne, Australia) for the nickel skimmer cone was used to increase the sensitivity of the instrument. The radio frequency (RF) generator power was set to 1400 W, and the extraction lens 1 and 2 voltages were 0 V and −220 V, respectively. The interface pressure was 1.7 mbar. Argon (5.0, Messer Industriegase GmbH, Siegen, Germany) was used as a nebulizer, plasma, and cooling gas with flow rates of 0.51, 0.8, and 14 L min^−1^, respectively. All dilutions were prepared in deionized, bi-distilled water. For ICP-MS determination of the silver and zirconium content in the immersion liquid of each zirconia substrate, 2 mL of the immersion liquid were added to 8 mL of 2% nitric acid (diluted from 70% nitric acid, Fisher Scientific, Loughborough, UK), respectively, and spiked with indium solution (Inorganic Ventures, Christiansburg, VA, USA) to obtain a concentration of 10 µg L^−1^ In as internal standard. For analysis, ion signals from ^90^Zr^+^, ^91^Zr^+^, ^107^Ag^+^, ^109^Ag^+^, and ^115^In^+^ were acquired in triplicate for each sample. Because the AgNP adhered to zirconia substrates, it was assumed that the zirconium concentration in the immersion liquid was high enough to cause significant signal interferences on the silver ion traces during ICP-MS analysis. Therefore, two separate dilution series of silver and zirconium stock solutions (Inorganic Ventures, Christiansburg, VA, USA), respectively, were prepared for silver concentration calibration and mathematical correction of the polyatomic interferences of ^91^Zr^16^O^+^ on ^107^Ag^+^ and ^92^Zr^16^O^1^H^+^ on ^109^Ag^+^.

### Antibacterial studies

Media and buffers have been prepared with Milli-Q water (Millipore Elix® Advantage 3, Millipak® Filter) and were sterilized *via* autoclaving (121 °C, 1.2 bar for 15 min).

*Escherichia coli* (*E. coli*, NTCT 10418) and *Staphylococcus aureus* (*S. aureus*, ATCC 29213, purchased at DMSZ as DSM 2569, wound isolate, Methicillin sensitive *S. aureus*) glycerol stocks stored at −80 °C were streaked onto LB (Lysogeny broth) agar (Carl Roth, Karlsruhe, Germany) and incubated at 37 °C until visible colonies were grown. Overnight cultures were prepared by inoculating 5 mL of LB (Luria/Miller: 10 g L^−1^ tryptone, 5 g L^−1^ yeast extract, 10 g L^−1^ NaCl, pH 7.0 ± 0.2) with a single colony and incubated at 200 rpm and 37 °C (incubator MaxQ6000, Fisher Scientific, Hampton, NH, USA) for 16 h to 18 h.

Overnight cultures were set to an OD_600_ of 0.5 in LB in semi-micro cuvettes (1.6 mL Rotilabo single-use, Carl Roth) and diluted 10-fold in LB. 1 mL of the cultures were then centrifuged at 10 000 rpm for 10 min at RT and the pellet was washed 2 times with PBS (phosphate-buffered saline, without calcium or magnesium; Lonza Walkersville, MD USA) and finally resuspended in 1 mL PBS. A further 100-fold dilution of the bacteria suspension in PBS resulted in a starting bacterial inoculum of 5.9 ± 0.2 log_10_ CFU mL^−1^ for *S. aureus* and 5.3 ± 0.1 log_10_ CFU mL^−1^ for *E. coli*.

Zr substrates (1 cm^2^ of anodized area) were sterilized in 2 mL 70% ethanol for 15 min in a 6 well culture plate (Sarstedt, Germany) and washed 3 times in 2 mL PBS before 2 mL of the starting bacteria inoculum were added. The plate was incubated at 37 °C for 24 h.

After incubation, bacterial culture suspensions in each well were collected, and the substrates were rinsed thrice with PBS. Non-adherent bacteria from each washing step were also collected.

To determine CFU mL^−1^ for each condition, a serial dilution (10-fold) in PBS was performed and spotted onto LB agar at least 3 times. LB agar plates were incubated for 24 h before colonies were counted. The log reduction factor (LRF) was determined by subtracting the average log_10_ CFU mL^−1^ of the treatment condition (Zr-NT, 24 h; Zr-NT/Ag-NP, 24 h) from the average log_10_ CFU mL^−1^ of the bacterial starting inoculum.

## Results and discussion

### Fabrication and characterization

**Fig. 1:**
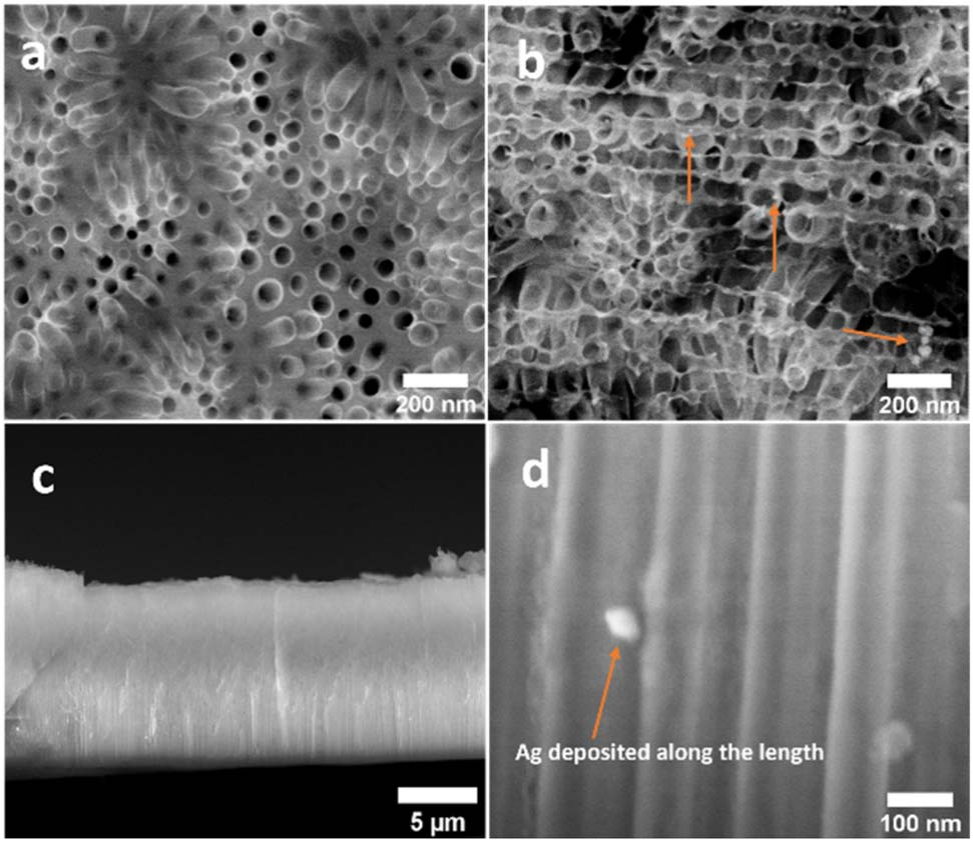
SEM top view image (a) zirconia nanotubes (ZrNTs) (fabricated by anodizing Zr metal) (b) ZrNT/AgNP composite material, the arrow indicates the location of the AgNP (fabricated by anodizing Ag–Zr alloy metal), (c and d) SEM cross-sectional morphology image, (c) side view of ZrNT/AgNP composite material, (d) high magnification of the side view, the arrow shows that AgNP were deposited within the nanotube along its length.

**Fig. 2:**
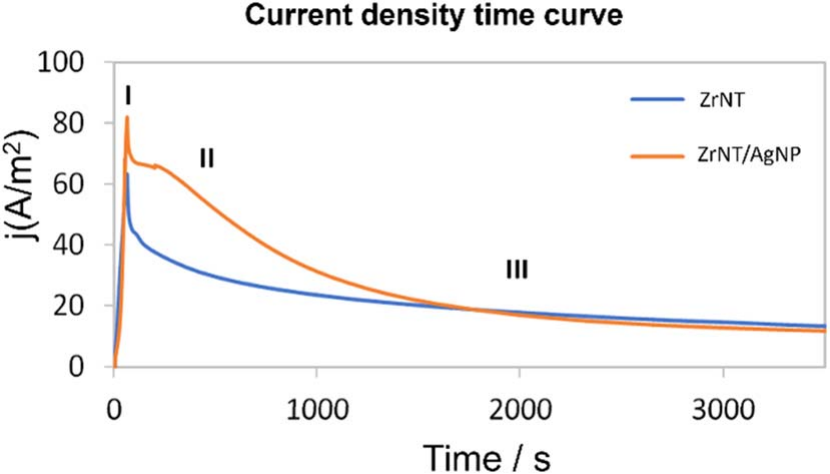
Comparison of the current density time curve during anodization for Zr (ZrNT) and Ag–Zr alloy (ZrNT/AgNP).

Alloy anodization was chosen as a method to produce AgNP-decorated ZrNTs in a one-pot approach.^56^ The fabricated ZrNT/AgNP composite and unmodified ZrNTs prepared by anodizing Zr–Ag alloy and pure Zr foil, respectively, were characterized using SEM to determine nanotube characteristics, such as diameter, length, surface coverage, and cross-sectional morphologies. Fig. 1a and b shows the top view of the fabricated nanostructure; from the top, the nanotubular structure is highly ordered, packed, and shows an average diameter of 80 ± 10 nm and 9 µm length for both samples, indicating that the overall anodization process remains unaltered due to the presence of Ag in the alloy. Alloy anodization, however, caused AgNPs to be uniformly deposited on the surface of the nanotubes and along the length of the nanostructure, as shown in Fig. 1c and d. Another observable difference is the presence of a thin mesh-like film of Ag_2_O sitting on top of the nanotubes for the anodized alloy, *cf.*Fig. 1d and SI 1. The electrochemical anodization of zirconium in a fluoride-containing electrolyte at optimal anodic conditions, *i.e.*, an appropriate value of voltage, temperature, pH, water content, *etc.*, is associated with the competing process between anodic oxidation of the Zr metal and dissolution of the metal oxide, resulting in uniform ZrNTs arrays. There are three main stages in the fabrication of the nanotubes.^57,58^ In the initial step, there is the oxidation of the zirconium metal to form a compact oxide barrier, and this barrier inhibits the further movement of ions, which is evidenced in the reduction of the current density. Subsequently, the oxide layer undergoes field-assisted dissolution and chemical dissolution due to the formation of a soluble zirconium-fluoro complex that allows for porosification. These pores serve as nucleation sites for the inward formation of porous ordered anodic oxide. In the third stage, when an equilibrium is established between the rate of oxidation and dissolution, stable nanotubular arrays are formed.^59,60^ These processes can be monitored by measuring the current density–time curve during the anodization, as shown in Fig. 2. The shape of the curve is similar for both sets of samples, indicating that the doping with Ag did not impede the nanotube formation, which is further evident in the similar nanotube dimensions as observed in Fig. 1. This result agrees with the work of Mazierski *et al.*^61^ where similar conclusions were made after anodizing different concentrations of silver-doped alloys of titanium. Gao *et al.*^62^ also reported little influence on the geometry, specifically, the diameter of the titania nanotube when they anodized AgTi coatings deposited by magnetron sputtering on Ti. However, the difference between the samples is the magnitude of the current density, and that can explain the formation of Ag_2_O and Ag NP in addition to the ZrNTs for the modified sample. The CD (current density), which is a function of the amount of moving ions in response to the applied potential, is slightly higher for ZrNT/AgNP (80 A m^−2^ compared to 60 A m^−2^ for unmodified ZrNTs), indicating enhanced dissolution of the oxide layer.^57,63^ The higher current densities may be explained by the enhanced conductivity of the ZrNTs due to the incorporated Ag. The formation of AgNP is explained by the electrochemical reduction of Ag^+^ to metallic Ag^0^, since the reduction potential of Ag^+^/Ag^0^ equals *E*° = 0.7994 V,^61^ in accordance with previous reports on Ti–Au alloy anodization.^56^

**Fig. 3:**
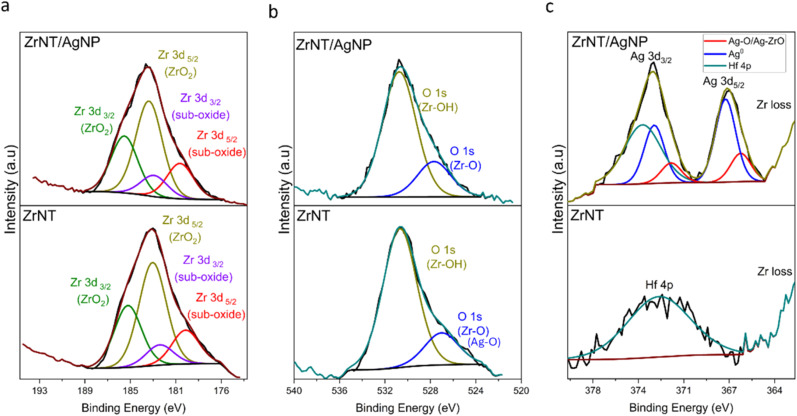
XPS measurement; high-resolution spectra on zirconia nanotubes (ZrNTs) and ZrNT/AgNP (a) Zr 3d (b) O 1s (c) Ag 3d.

EDX, coupled with SEM, was used to determine the presence and Ag content of the composite material; results are summarized in Table 1 and describe the average elemental percentage, calculated from EDX analysis of 3 different spots of the anodized area. The average weight percentage of Ag is 1.5 ± 0.8%, and this value agrees quite favorably with the actual weight percentage of Ag in the original Zr–Ag alloy, indicating that Ag is not lost in the production process. As the information depth of EDX typically exceeds the layer thickness of anodic nanostructures, XPS analysis was performed to obtain information about the surface composition. A general XPS survey (Fig. SI 2) compares the chemical composition of the different samples, and the expected peaks of Zr 3p/3d and O 1s were observed for both samples. The peaks of F 1s and C 1s are caused by the organic electrolyte used in the anodization. Despite an attempt at removing this residue by extensive rinsing in ethanol, there was still an observable quantity in the nanotube matrix, potentially incorporated into the tube walls (*cf.* Table SI 1). These residues can be removed from the nanotubes by an annealing treatment if required for the desired application, which is commonly necessary for using anodic oxide nanostructures in catalysis or as electrodes.^64^ As this step poses a risk of AgNP aggregation and the electronic properties of the material are secondary for the desired application, it was omitted in this project. High-resolution XPS analysis (Fig. 3c) of the Ag 3d region confirms the presence of Ag on the ZrNTs. The signal at 368.2 eV confirms the presence of metallic Ag^0^. The two distinct signals of Ag 3d_3/2_ and Ag 3d_5/2_ at 373.8 eV and 367.2 eV, respectively are indicative of Ag^+^ species, and correlate with binding energies reported for Ag_2_O.^65,66^ This indicates that the silver in the tube-top region of the ZrNTs is present in both oxidized form and as metallic Ag^0^. The signal observed on the pure ZrNTs sample corresponds to Hf 4p,^67^ as the zirconium foil used also contains hafnium, which is part of the base metal. The high-resolution XPS analysis of the Zr 3d region shows the characteristic doublet peaks of 3d_5/2_ and 3d_3/2_ at higher binding energy (compared to metallic Zr (Zr^0^), indicating the presence of oxidized zirconium (Zr^4+^)).^67^ The binding energies of 3d_3/2_ and 3d_5/2_ are at 185.4 eV and 182.7 eV for ZrNT/AgNP, respectively; and 184.9 eV and 183.2 eV for ZrNTs, respectively. A slight chemical shift of the 3d peaks was observed, suggesting a possible loss of electron density around the Zr atom due to the presence of Ag in the ZrNT/AgNP substrate.^68^ Additionally, suboxides at lower binding energies were detected in both substrates. This observation is consistent with previous reports that oxidized zirconium can form intermediate suboxide species in addition to fully oxidized ones.^69^ The deconvoluted O 1s peak shows two signals at the binding energy of 530.7 eV and 527.7 eV on the ZrNT/AgNP substrate, and 530.5 eV and 527.1 eV for the ZrNTs substrate. Both substrates have similar binding energy, indicating lattice oxygen coordination within the ZrO_2_ crystal lattice at the lower BE and a higher BE, which correspond to adsorbed hydroxide group (–OH) on the zirconia surface. Zirconia readily absorbs water from the atmosphere, leading to the formation of Zr–OH.^70^

**Table 1:** Summarized EDS measurement on ZrNT/AgNP composite material showing the weight and atomic percent of the relevant elements

	wt%	at%
O	22.94	45.15
F	21.53	35.69
Zr	53.25	18.78
Ag	1.50	0.38

**Fig. 4:**
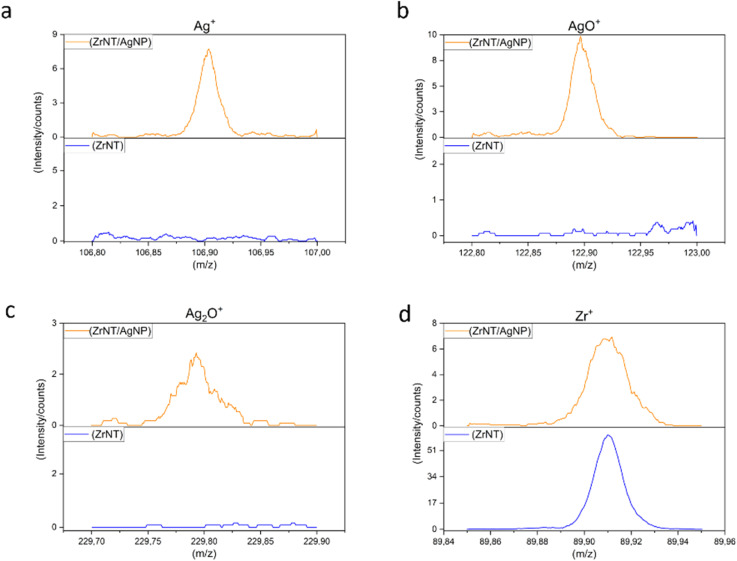
ToF – SIMS molecular fragment of (a) Ag^+^, (b) AgO^+^, (c) Ag_2_O^+^, (d) Zr^+^ (substrate signal).

Additionally, ToF-SIMS measurements were conducted to complement the results from EDX and XPS. ToF-SIMS has the highest sensitivity (ppm) of the three techniques, combined with a shallow information depth of 1-3 nm. Signals observed at *m*/*z* of 106.9 and 122.9 are indicative of ^107^Ag^+^ and its oxide ion fragment ^107^Ag^16^O^+^, respectively. As observed in Fig. 4, the intensity of Ag-related signals is significantly higher for the ZrNT/AgNP. This indicates that at least a fraction of the AgNPs is oxidized or bonded to the ZrNTs in Ag–O–Zr bonds, consistent with XPS.

**Fig. 5:**
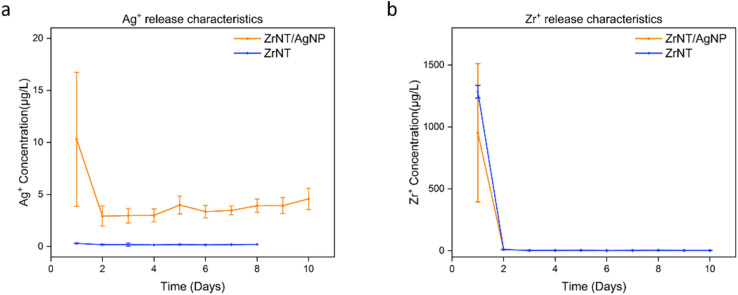
ICP-MS measurement monitoring the ion release behavior of (a) Ag^+^ and (b) Zr^+^ for both samples (3 replicates were analyzed for each sample).

The efficacy of incorporated Ag as an antibacterial agent is related to the release of Ag in its oxidized dissolved form (*i.e.*, as Ag^+^).^71,72^ Since the ultimate goal is to design metallic implant surfaces with Ag-induced surface reactivity, it is important to investigate the release of Ag ions from the fabricated samples in aqueous media to determine the extent of AgNPs releasing Ag^+^ ions for effective antibacterial activity. The latter amount was determined by ICP-MS; the release of Zr ions was additionally monitored. Fig. 5 compares the Ag^+^ release behavior from both samples. As expected, no release of Ag^+^ could be observed for pure ZrNTs within the monitoring for 10 days. The anodized alloy, ZrNT/AgNP, demonstrated Ag^+^ release with an initially higher concentration (10 µg L^−1^) that stabilized to a constant amount of (∼4 µg L^−1^). In aqueous solutions, it has been reported that silver nanoparticles can be oxidized, and under acidic conditions, silver ions are released.^73^ This can explain the Ag release from the ZrNT/AgNP sample, where the AgNPs are oxidized (eqn (1)) and released into the PBS medium. During sample preparation for ICP-MS measurements, nitric acid is used as part of the dilution and conservation medium and provides the acidic conditions necessary for the dissolution of silver oxide to silver ions (eqn (2)).

1 4Ag^0^ + O_2_ → 2Ag_2_O

2 2Ag_2_O + 4H^+^ → 4Ag^+^ + 2H_2_O

The release pattern observed in Fig. 5 is consistent with release behavior from ordered nanotubular structures. At the initial stage, the PBS solution interacts with the AgNPs at the surface, and due to a higher concentration gradient, a burst release is triggered. Subsequently, equilibrium concentration is established as the solution gradually infiltrates along the nanotube, and a constant controlled amount is observed due to the homogenous distribution of the AgNPs and oxide in the nanotubular matrix. The toxicity of Ag ions to human cells is generally observed from above 1 mg L^−1^,^48^ the amount of Ag ions released from this system is between 10 µg L^−1^ to 4 µg L^−1^, significantly below the toxicity threshold. Furthermore, studies have shown that below 40 µg L^−1^ AgNP exhibits some favorable effects on promoting cell spreading.^62^ Therefore, this approach shows good promise for incorporating into medical devices aimed at enhancing interaction with human cells while maintaining long-term antibacterial activity.

### Antimicrobial tests

**Fig. 6:**
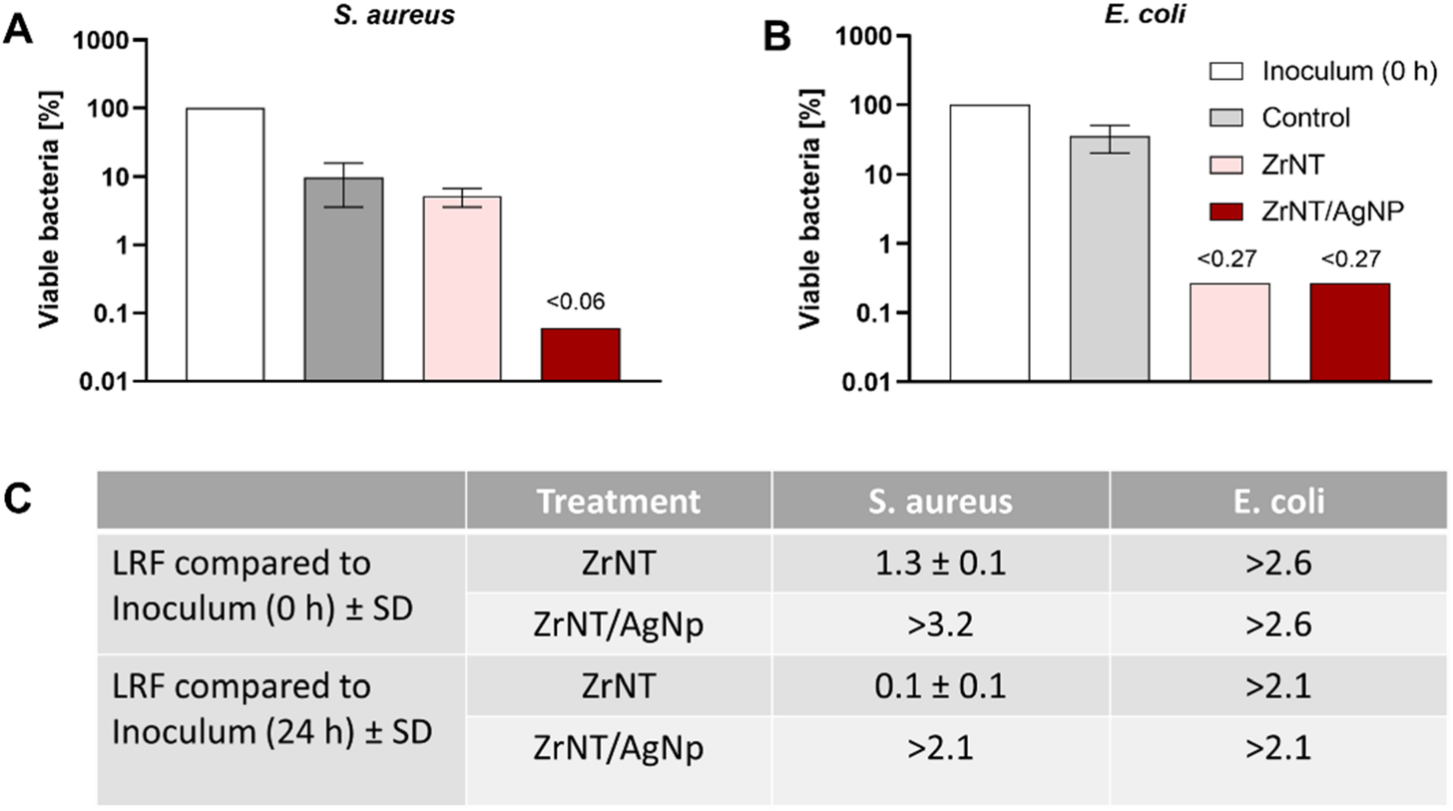
Antimicrobial activity of ZrNT and ZrNT/AgNP on (A) *S. aureus* ATCC 29213 and (B) *E. coli* NTCT 10418. Bacterial inocula at concentrations of (A) 5.9 ± 0.2 log_10_ CFU mL^−1^ and (B) 5.3 ± 0.1 log_10_ CFU mL^−1^ were exposed to ZrNTs and ZrNT/AgNp substrates for 24 h at 37 °C or cultivated untreated under control conditions in 6-well plates. A and B: Remaining viable bacteria after 24 h under different treatment conditions are indicated in % compared to the bacterial inoculum at timepoint 0 h, which was set to 100% (for absolute log_10_ CFU counts see Fig. SI 4). Considering the limit of detection for CFU, counting leads to a maximal (<) postulated 0.06% for *S. aureus* (A) or 0.27% for *E. coli* of viable bacteria, or (C) a minimal (>) postulated 3.2 and 2.6 log reduction factor (LRF) for *S. aureus* and *E. coli*, respectively. Error bars: standard deviation of 4 CFU-counting replicates.

The prepared ZrNT/AgNP and unmodified ZrNT substrates were tested for their antimicrobial properties as proof of concept towards common pathogens that often cause biomaterial-associated infections, namely *E. coli* and *S. aureus*, which serve as models for Gram-negative and Gram-positive bacteria, respectively, to demonstrate the broad-spectrum effectiveness of the modified biomaterial.^74,75^Fig. 6 and SI 3 illustrate that ZrNT/AgNP exhibits high antimicrobial activity against both *S. aureus* and *E. coli*. Against *S. aureus* bactericidal activity, which is defined as the killing of more than 99.9% bacteria of the initial inoculum, meaning a log reduction factor (LRF) > 3 was achieved, while for *E. coli*, an LRF > 2.1 was reached, meaning less than 0.270% viable bacteria of the starting inoculum could be detected. Unmodified ZrNT exhibited antimicrobial activity to the same extent (LRF > 2.6) against *E. coli* but not towards *S. aureus*, hinting at a clear difference in the antimicrobial efficiency and presumably the influencing mode of action of ZrNTs against Gram-positive compared to Gram-negative bacteria. Fig. SI 4 reveals that nanotube formation plays an essential role in the antimicrobial effect of both ZrNTs and ZrNT/AgNP, as the Zr-foil and the Zr–Ag alloy foil do not exhibit any antimicrobial effect, as is proven by control antimicrobial tests with just the corresponding foil. One has to consider that for *S. aureus*, incubation with PBS causes 1 LRF (Fig. 6 and SI 3). Therefore, the antimicrobial effect caused by the tested material, when comparing the log_10_CFU ml^−1^ with the control condition that was incubated for 24 h in PBS, was about 1 LRF (*S. aureus*) or 0.5 LRF (*E. coli*) lower for all treatment conditions than the LRF calculated relative to the starting inoculum (0 h). These results could all be validated through independent replication of the antibacterial tests using different substrate batches and bacterial cultures (Fig. SI 5). Slight variations in antimicrobial efficacy, indicated by the LRF, were observed to potentially be caused by small differences in the substrates and the anodization process or the starting bacterial inoculum.

The known antibacterial effect of silver-based materials has been extensively studied for a long time, and its activities have been reported to be entirely due to the release of silver ions and negligibly due to a direct influence from the shape or size of the particle.^72^ AgNP can serve as an effective vehicle to deliver Ag ions directly to the bacterial membrane and cytoplasm by evading binding with natural ligands (which reduces its bioavailability).^76^ Others showed that AgNP activity against *E. coli* and *S. aureus* decreases the activity of the respiratory chain dehydrogenase of both bacteria, enhances the protein and reduces sugar leakage from the bacterial membrane by increasing its permeability. It forms reactive oxygen species, which can damage the bacterial protein structure and its intracellular system.^77^ This also agrees with other published work that came to a similar conclusion that Ag acts through three main mechanisms, such as membrane damage, production of reactive oxygen species, and cellular uptake of silver ions.^78^

Antibacterial activity from the undecorated ZrNTs against *E. coli* observed can be due to two reasons: zirconia nanoparticles have been demonstrated to have antibacterial properties, primarily due to the interaction of the positively charged zirconium ions with negatively charged bacterial (*E. coli*) cell walls, which can potentially result in the rupturing of the bacterial cell walls and ultimately cell death.^79^ This holds true for the undecorated ZrNTs, and the release of zirconium ions was confirmed *via* ICP-MS, see Fig. 5. Secondly, recent studies have shown that nanoscale patterning can induce the behavior of bacteria on surfaces in terms of ordering and orientation; hence, even without decoration with an antibacterial agent, the geometrical morphology of the nanotubes can cause an antibacterial effect.^80,81^ This effect of “piercing nanostructures” was observed to be enhanced for Gram-negative bacteria.^82,83^ Thirdly, the presence of fluoride in the nanotube can also play a role in the antibacterial properties, especially against Gram-negative bacteria. However, fluoride ions are generally less effective against Gram-positive bacteria like *S. aureus* under neutral or basic conditions.^84^

The primary challenge in using silver nanoparticle coatings as an antibacterial agent in implant systems to reduce the incidence of biomaterial-associated infections has been the risk of toxicity to human cells, which is linked to the release behavior. In this work, it has been illustrated that a controlled release behavior with a minimal amount of Ag^+^ ions can be achieved *via* a simple, straightforward electrochemical anodization of a Zr–Ag alloy (the wt% of Ag remains constant during anodization). The antibacterial test showed an effective antibacterial activity against the Gram-positive and Gram-negative model bacteria *S. aureus* and *E. coli*, and for the first time, to the best of our knowledge, it was shown that ZrNTs themselves possess antibacterial properties against Gram-negative bacteria. In future work, the antimicrobial activity of the tested substrates will be screened for more bacterial species to prove if these antibacterial effects can be generalized for Gram-positive and Gram-negative bacteria. Additionally, the influence of the nanostructural morphology on the antibacterial activity, as well as the impact of Ag decoration on the integration with human tissues, will be investigated in-depth. Finally, further evaluation *in vivo* is required to assess its performance under the increased complexity of biological conditions.

## Conclusions

A simple strategy for improving the antibacterial properties of ZrNTs *via* decoration with AgNP that consist partially of Ag_2_O was described. This involves the electrochemical anodization of a Zr–Ag alloy to form a ZrNT/AgNP composite material embedded with AgNP along the length of the nanotube while also preserving the nanotubular morphology that can promote cellular integration and potentially be modified for multifunctionality. The composite nanomaterial was found to be an effective antibacterial agent against *Escherichia coli* and *Staphylococcus aureus*, killing more than 99.9% of bacteria of the initial inoculum after 1 day, with 10 µg L^−1^ of silver ion release. The Ag ion release was at a controlled and relatively constant rate for the 10 days monitored. ZrNTs showed antibacterial activity against *Escherichia coli* even without any modification, and this indicates the activity of zirconium ions and/or the impact of nanotubular topography, especially against Gram-negative bacteria, which might even act synergistically with the effect of silver ions. Conclusively, it has been demonstrated that ZrNTs intrinsically decorated with AgNP during anodization can be a promising candidate as a coating on implant material in preventing the incidence of biomaterial-related infections.

## Conflicts of interest

The authors report no conflicts of interest.

## Supplementary Material

RA-016-D5RA07099F-s001

## Data Availability

Data for this article, including SEM images, processed XPS and ToF-SIMS spectra, ICP-MS results, antibacterial study data, are available at Zenodo at (https://doi.org/10.5281/zenodo.16833021). Supplementary information (SI): additional SEM images comparing the top-view morphology of the fabricated zirconia nanotubes, highlighting differences due to silver modification. It also includes XPS survey data of the nanotubes surfaces and further results from the antibacterial studies. See DOI: https://doi.org/10.1039/d5ra07099f.
