# Supplementary material for: Novel Ag-modified zirconia nanomaterials with antibacterial activity
Source: RSC Adv. 2026 Jan 9;16(3):2286–97. doi: 10.1039/d5ra07099f (PMC12784246; doi:10.1039/d5ra07099f)
Supplement: RA-016-D5RA07099F-s001 [file RA-016-D5RA07099F-s001.pdf]

Supplementary Information (SI) for RSC Advances.
This journal is © The Royal Society of Chemistry 2025

Supplementary material

Novel Ag-modified zirconia nanomaterials with antibacterial activity

Gabriel Onyenso[a,e], Jiwar Al-Zawity[b,e], Nastaran Farahbakhsh[a,e], Annika Schardt[c,e], Aydan Yadigarli[a,e], Swathi Naidu Vakamulla Raghu[a,e], Carsten Engelhard [c,d,e], Mareike Müller[b,e], Holger Schönherr[b,e], Manuela S. Killian[a,e]

(a) Chemistry and Structure of Novel Materials, University of Siegen, Paul-Bonatz-Str. 9-11, 57076 Siegen, Germany

(b) Physical Chemistry I, University of Siegen, Adolf-Reichwein-Str. 2, 57076 Siegen, Germany

(c) Analytical Chemistry, University of Siegen, Adolf-Reichwein-Str. 2, 57076 Siegen, Germany.

(d) Federal Institute for Materials Research and Testing (BAM), Richard-Willstätter Str. 11, D-12489 Berlin, Germany

(e) Research Center of Micro- and Nanochemistry and (Bio)Technology (Cμ), University of Siegen, Germany

KEYWORDS: oxide nanostructures, zirconia, antibacterial coatings, silver nanoparticles

ORCID: G. Onyenso: 0009-0003-5140-0055, J. Al-Zawity: 0009-0006-7873-2377, N. Farahbakhsh: 0000-0002-0136-7416, A. Schardt: 0000-0002-8308-3686, A. Yadigarli: 0000-0002-8824-7619, S.N.V. Raghu: 0000-0001-8960-9581, C. Engelhard: 0000-0002-7020-9278, M. Müller: 0000-0003-0891-5160, H. Schönherr: 0000-0002-5836-5569, M.S. Killian: 0000-0003-0892-4614

Electrochemical anodization of Zr and Ag-Zr alloy

Anodization of zirconium in a fluoride-containing electrolyte at suitable conditions, such as voltage, temperature, pH, etc, leads to the formation of zirconia nanotubes (ZrNTs). However, when doped with other metals like Ag, there is the possibility to chemically and structurally modify the ZrNTs to achieve specific properties. The Figure below compares the top view of Zr and Ag-Zr alloy foil (~ 1wt% Ag) after anodizing at a constant 90 V for 1 h, respectively (left to right). For the modified ZrNTs, in addition to the nanotube, AgNp, and a mesh of possible silver oxide were observed. It is critical to observe that the silver oxide did not cover the nanotubes; hence, they can be loaded with other molecules to achieve multifunctionality.

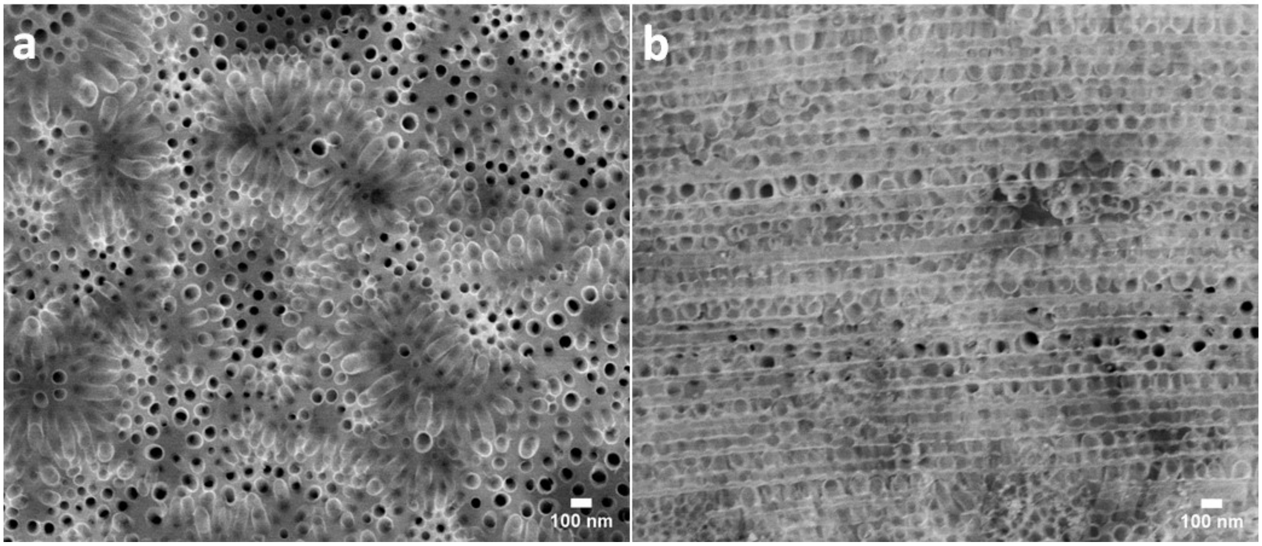

Figure SI 1: SEM top view image a) ZrNTs b) ZrNT/Ag_2O/AgNP composite material.

XPS measurement

The figure below shows the X-ray photoelectron spectroscopy (XPS) survey of the ZrNTs and ZrNT/AgNP, indicating the elemental compositions on the surface of the samples. Table S1 depicts the atomic percentages of all observed elements.

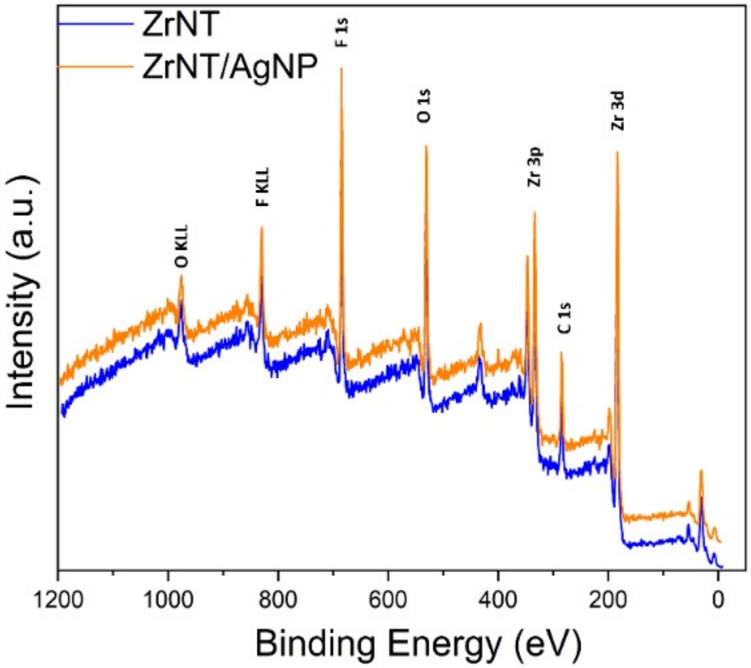

Figure SI 2: XPS measurement; *survey of ZrNTs and ZrNT/AgNP.*

Table SI 1: XPS measurement; *at% values of all detected elements*

Sample	Atomic percent					
	F 1s	C 1s	O 1s	Zr 3d	Ag 3d	Hf 4p
ZrNT	25.3	26.8	29.2	18.0	-	0.7
ZrNT-Ag	23.8	27.0	29.7	18.1	0.9	0.5

Antimicrobial test

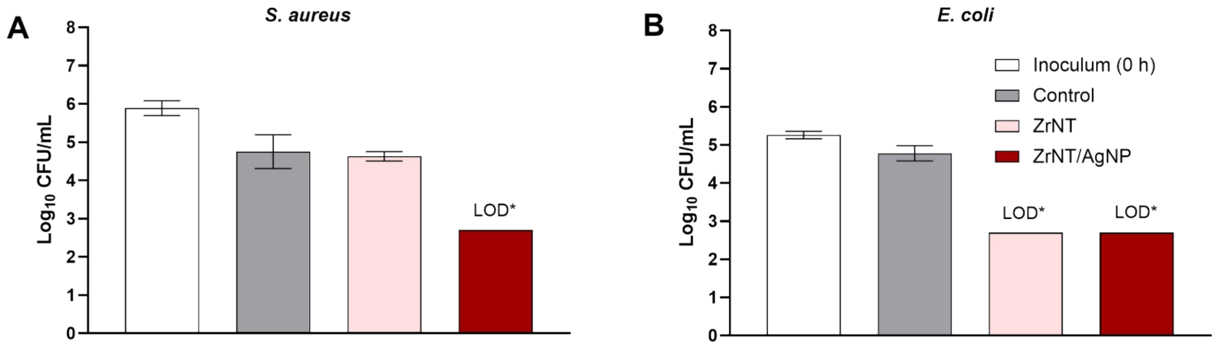

Figure SI 3: Reduction of log_{10} CFU/mL of (A) S. aureus ATCC 29213 and (B) E. coli NTCT 10418. Bacterial inocula (0 h) at concentrations of (A) 5.9 +/- 0.2 log_{10} CFU/mL and (B) 5.3 +/- 0.1 log_{10} CFU/mL were exposed to ZrNTs and ZrNT/AgNP substrates or cultivated untreated under control conditions for 24 h at 37°C in 6-well plates. LOD: The limit of detection for CFUs counting was 500 CFU/mL. Error bars: Standard deviation of 4 CFU-counting replicates.*

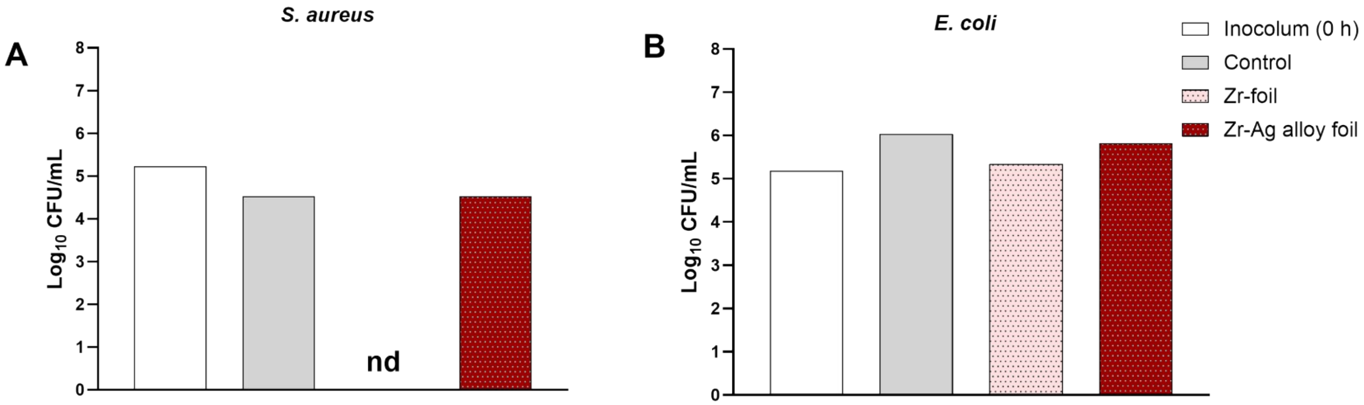

Figure SI 4: Zr and Zr-Ag alloy foil do not exhibit any antimicrobial property. Bacterial inocula of S. aureus ATCC 29213 and E. coli NTCT 10418 (0 h) were exposed to Zr-foil or Zr-Ag alloy foil or cultivated untreated under control conditions *for 24 h at 37°C in* 6-well *plates. Resulting log_{10} CFU/mL determined with CFU-counting are blotted. There is no relevant antimicrobial effect detected as all LRF are below 1. Nd: not defined.*

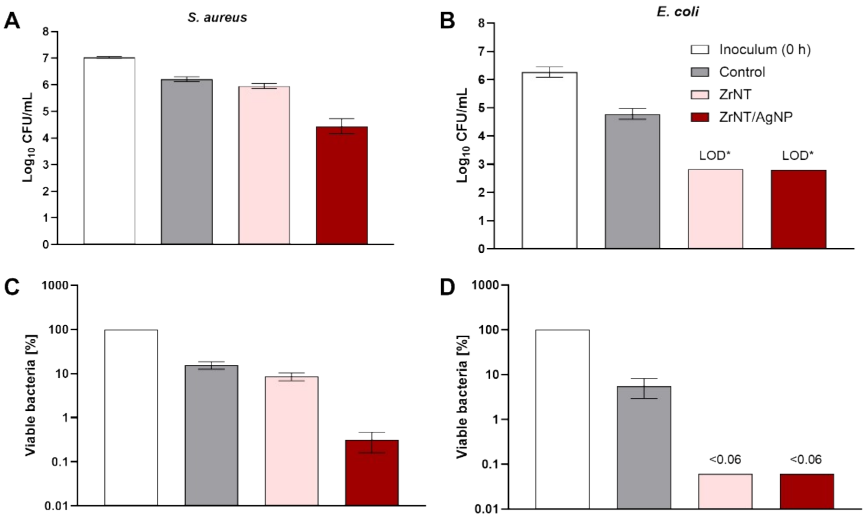

E

	Treatment	*S. aureus*	*E. coli*
LRF compared to Inoculum (0 h) ± SD	ZrNT	1.1 ± 0.1	>3.4
	ZrNT/AgNP	2.6 ± 0.3	>3.4
LRF compared to Inoculum (24 h) ± SD	ZrNT	0.3 ± 0.3	>2.0
	ZrNT/AgNP	1.8 ± 0.3	>2.0

Figure SI 5: Antimicrobial activity of ZrNTs and ZrNT/AgNp on (A,C) S. aureus ATCC 29213 and E. coli NTCT 10418 (B,D) (reproduction of results from Figure 6 in a completely independent experiment). Bacterial inocula (0 h) were exposed to ZrNTs and ZrNT/Ag-Np/AgO substrates for 24 h at 37°C or cultivated untreated under control conditions in 6 well plates. A and B: Reduction of absolutely counted log_{10} CFU/mL before and after treatment is shown. LOD: The limit of detection for CFUs counting was 667 CFU/mL. C and D: Remaining viable bacteria after 24 h under different treatment conditions are indicated in % compared to the bacterial inoculum at timepoint 0 h, which was set to 100 %. Considering the limit of detection for CFU counting leads to a maximal (<) postulated 0.06 % (D) or (C) a minimal (>) postulated 3.4 log reduction factor (LRF) for E. coli. Error bars: Standard deviation of 3 CFU-counting replicates.*
